# Resilience and its influencing factors after emergency percutaneous coronary intervention in young and middle-aged patients with first acute myocardial infarction

**DOI:** 10.1038/s41598-024-59885-9

**Published:** 2024-04-25

**Authors:** Jinju Wang, Yafeng Wu, Juanjuan Zhou, Shaoman Li, Liping She

**Affiliations:** Department of Cardiology, Nanjing First Hospital, Nanjing Medical University, 68 Changle Road, Nanjing, 210006 Jiangsu China

**Keywords:** Resilience, Myocardial infarction, Young and middle-aged, Influencing factors, Emergency PCI, Public health, Population screening

## Abstract

Mental health after acute myocardial infarction (AMI) influences the prognosis of patients. Resilience may contribute to improving a patient’s mental health. However, no study has investigated resilience and its associated factors in young and middle-aged patients undergoing emergency percutaneous coronary intervention (PCI) after the first AMI. This study aimed to identify critical associated factors influencing resilience in these patients. This cross-sectional study recruited 161 young and middle-aged patients with first-episode AMI using a purposive sampling method. These patients were assessed 48 h after emergency PCI using the General Information Questionnaire, the Connor—Davidson Resilience Scale—10, the Perceived Social Support Scale, the General Self-Efficacy Scale, and the Post-traumatic Stress Disorder Scale Civilian Version. Stepwise and logistic regression were conducted to analyze the factors influencing resilience. Receiver operating characteristics (ROC) were used to compare the area under the curves (AUC) for each indicator. The resilience of the 161 participants was 29.50 ± 4.158. Monthly household income, self-efficacy, social support, and post-traumatic stress disorder explained 51.4% of the variance in resilience. Self-efficacy (*OR* 0.716, CI 0.589–0.870, *P* < 0.01) and social support (OR 0.772, CI 0.635–0.938, *P* < 0.01) were protective factors for psychological resilience, while post-traumatic stress disorder (OR 1.278, CI 1.077–1.515, *P* < 0.01) was a risk factor. ROC curve revealed that self-efficacy, social support, and PTSD had an AUC of 0.822, 0.855, and 0.889, respectively. Self-efficacy and social support improve, and PTSD degrades psychological resilience in young and middle-aged AMI patients undergoing emergency PCI.

## Introduction

Acute myocardial infarction (AMI) is the leading cause of death resulting from cardiovascular diseases worldwide, affecting approximately one million people in China annually^1^. Contrary to popular belief, the incidence of AMI is not limited to elderly patients; recent studies have shown a gradual increase in AMI incidence in younger individuals^2^. The China Cardiovascular Health Report indicated a 36% increase in AMI incidence among young individuals aged 25–44 from 1990 to 2019, with about one-third of acute AMI patients younger than 60^3^.

Emergency percutaneous coronary intervention (PCI)^4^ has become increasingly popular for treating AMI patients. However, the invasive nature of the procedure and permanent stent placement can cause various psychological problems in patients, including anxiety, depression, and post-traumatic stress disorder (PTSD) associated with poor prognosis and high mortality^5–7^. These psychological responses may be more significant in emergency PCI settings, and this psychological pressure may prevent successful return reintegration into society and family life^8^.

Resilience is the ability of an individual to recover from or rebound from adversity^9^, and it is proportional to mental health and life satisfaction, mitigating the adverse effects of stress^10^. However, studies on resilience in AMI patients are limited. Previous research looked into the relationship between resilience, self-efficacy, and negative affect in AMI patients after PCI and have confirmed that resilience and PTSD are correlated^11,12^. Young and middle-aged patients are critical for social development and family life. However, Studies have shown that anxiety is significantly higher among young and middle-aged patients than among elderly patients^13^, they have multiple responsibilities and stressors, which cause them to suffer from impaired cardiac function after emergency PCI, such as angina pectoris and heart failure, resulting in limited physical activity^14^ and the ability to work and socialize. The disease has a significantly greater impact on their lives and the economy than on other age groups^15^. According to Kumpfer’s resilience theory, resilience can mediate the relationship between adversity and its outcomes and propel a person to grow in the face of adversity^16^. Most previous studies on resilience have focused on patients aged 18–80 years undergoing elective PCI. Few studies have focused on resilience and its influencing factors in young and middle-aged patients undergoing emergency PCI for their first AMI. Understanding such patients’ resilience and the impact factors may prevent and treat psychological problems and promote disease recovery and physical and mental health.

Therefore, this study aimed to assess the resilience status of young and middle-aged patients who had the first AMI after emergency PCI and investigate the relationship between sociodemographics, self-efficacy, social support, and PTSD and resilience. In addition, this study evaluated the predictive value of these factors in predicting low resilience using the receiver operating curve (ROC).

## Methods

### Participants

Purposive sampling was used in this cross-sectional study by recruiting 161 AMI patients between May 2020 and February 2022 from the Coronary Care Unit (CCU) Cardiology Department, Nanjing Hospital, Nanjing Medical University. The inclusion criteria of patients were:

1. 18–60 years old.
2. Diagnosis meeting the Chinese Medical Association’s revised Acute ST-segment elevation myocardial infarction diagnostic criteria.
3. Symptoms of AMI on admission but never before.
4. Unaware of the presence of coronary artery disease before admission for AMI.
5. Admitted via emergency and received first emergency PCI.

Patients who were critically ill, had cognitive impairment, vital organ dysfunction, hematological diseases, and malignant tumors were excluded.

### Calculation of sample size

The researchers used PASS 15.0 software to precalculate the sample size with a 95% confidence interval, 30% overall standard deviation^17^, and 0.15 tolerance. The computation produced 155 cases, the bare minimum sample size needed. In the end, 161 patients were included.

### Data collection

This study collected data within 48 h of the patient’s mental status and condition stabilizing after emergency PCI. After all participants provided written informed consent, they received a questionnaire designed by the researcher. If patients were unable to complete the questionnaire on their own, the researcher read aloud the questions and recorded the patients’ responses on their behalf.

### Ethical consideration

The Ethics Committee of Nanjing Hospital of Nanjing Medical University approved the study (Approval No., KY20200424-03.), and all methods were performed in accordance with the Declaration of Helsinki. Before the questionnaire began, participants signed informed consent forms.

### Instruments

#### Demographic variables

After reviewing the literature, the researcher developed his own demographic questionnaire^18^ (Supplementary file 1), which included age, gender, presence of a spouse, education level, work status, nature of work, payment of medical expenses, residence status, place of residence, and monthly income level.

#### Connor–Davidson resilience scale 10 (CD-RISC-10)

The Conner–Davidson resilience scale (CD-RISC) was used to develop the CD-RISC-10 by Campbell-Sills and Stein (2007), and Zengjie Ye et al. (2018) translated the Chinese version to measure resilience among adults^19^. It comprises 10 items scored on a 5-point Likert scale, ranging from 0 (very non-conforming) to 4 (very conforming) with a total score of 0–40; higher scores imply higher levels of psychological resilience. The CD-RISC-10 has superior psychometric properties, reliability, and applicability to Chinese people compared to the CD-RISC scale^20,21^. The scale’s Cronbach’s alpha coefficient in this study was 0.922.

#### General self-efficacy scale (GSES)

Schwarzer^22^ developed the GSES to assess an individual’s ability to cope with different environments and their general self-efficacy when facing new challenges. The Chinese version was translated and corrected by Wang et al.^23^.

Scores are based on a Likert 4-point scale, where 1 represents completely incorrect, and 4 represents completely correct. Scores range between 10 and 40; higher scores indicate stronger self-efficacy. The Chinese version of the GSES has proven to be highly reliable and valid^24^. The scale’s Cronbach’s alpha coefficient in this study was 0.87.

#### Perceived social support scale (PSSS)

The PSSS was developed by Zimet et al.^25^ and translated into Chinese by Qianjin^26^ to measure individual sources of social support. The scale has three components: support from friends, family, and other sources, with 12 items scored on a Likert scale with seven points ranging from 1 (strongly disagree) to 7 (strongly agree). The overall score was 12–84, with higher scores indicating stronger social support for their apprehension. The scale’s Cronbach’s alpha coefficient in this study was 0.943.

#### The PTSD check list-civilian version (PCL-C)

The PCL-C scale was developed from the DSM-W^27^ to evaluate the experiences of common people following traumatic experiences in ordinary life. The 17-item scale includes three dimensions of re-experience, avoidance or numbing, and hyperarousal, scored on a 5-point scale, ranging from 1 (did not occur) to 5 (extremely severe). The total score range was 17–85. This study conducted an extensive literature review to establish a diagnostic threshold of 38 for PTSD in Chinese individuals to increase its diagnostic validity^28^. Patients with a total score of ≤ 37, 38–49, and ≥ 50 represented no significant PTSD symptoms, some degree of PTSD symptoms, and substantial PTSD symptoms, respectively. Item score ≥ 3 was considered a positive item. The number of positive items of re-experiencing symptoms, avoidance/numbness symptoms, and hypervigilance symptoms were ≥ 1, ≥ 3, and ≥ 2, respectively. The Cronbach’s alpha coefficient for this scale was 0.94.

### Statistical analysis

The data was analyzed using IBM SPSS Statistics (version 26.0) statistical software. *P* < 0.05 was regarded as statistically significant. GraphPad Prism (version 9.0) software was used to plot the ROC curves. In this study, means, standard deviation, and frequency (percentage) were used to describe continuous and categorical variables, to make a statistical description of baseline characteristics and resilience scores of the study population. Normally distributed variable scores were tested using *t*-tests and one-way ANOVA or chi-square analysis. Pearson or Spearman correlation coefficient (*r*) was used to examine the relationships between resilience and sociodemographics, general self-efficacy, social support, and post-traumatic stress disorder. In addition, stepwise multiple linear regression and univariate and multivariate logistic regression analyses were used to determine the factors influencing resilience. The ROC was used to assess the predictive value of different variables on resilience and to determine the optimal value.

## Results

### Participants characteristics

This study included 161 participants with an age range of 33–60 years. In addition, 85.8% of participants were aged 45–60 years, and the majority (91.3%) were male (Table 1).

**Table 1:** Demographics of participants (n = 161).

Variables	N (%)	Resilience			High resilience (N = 81)	Low resilience (N = 80)	χ^2^/T	p
		Mean (SD)	T/F	P				
Age			0.383	0.702	51.17 ± 6.11	52.19 ± 5.81	− 1.08	0.282
< 45 years	22 (13.6%)	29.82 ± 4.04						
45–60 years	139 (85.8%)	29.45 ± 4.17						
Gender			− 0.603	0.547			1.198	0.274
Male	147 (91.3%)	29.44 ± 4.13			72 (88.89)	75 (93.75)		
Female	14 (8.7%)	30.14 ± 4.35			9 (11.11)	5 (6.25)		
Marriage			0.705	0.482			3.009	0.083
Single/divorced	9 (5.59%)	28.56 ± 3.40			2 (2.47)	7 (8.75)		
Married	152 (94.41%)	29.56 ± 4.19			79 (97.53)	73 (91.25)		
Education			3.96	0.021*			6.456	0.040*
Primary school	37 (22.98%)	28.78 ± 3.73			14 (17.28)	23 (28.75)		
High or secondary school	105 (65.22%)	29.32 ± 4.27			53 (65.43)	52 (65.00)		
College or university	19 (11.8%)	31.89 ± 3.51			14 (17.28)	5 (6.25)		
Monthly household income			7.643	0.001**			7.377	0.025*
< 5000	23 (14.29%)	29.04 ± 4.51			10 (12.35)	13 (16.25)		
5000–10,000	99 (61.49%)	28.76 ± 3.93			44 (54.32)	55 (68.75)		
> 10,000	39 (24.22%)	31.67 ± 3.78			27 (33.33)	12 (15.00)		
Residence			2.533	0.083			3.19	0.203
City	138 (85.71%)	29.40 ± 4.08			69 (85.19)	69 (86.25)		
Town	6 (3.73%)	33.17 ± 4.02			5 (6.17)	1 (1.25)		
Countryside	17 (10.56%)	29.06 ± 4.37			7 (8.64)	10 (12.50)		
State of residence			− 1.888	0.061			5.044	0.025*
Living with others	137 (85.09%)	29.76 ± 4.04			74 (91.36)	63 (78.75)		
Living alone	24 (14.91%)	28.04 ± 4.53			7 (8.64)	17 (21.25)		
Working status			1.22	0.224			1.441	0.23
Regular employee	140 (86.96%)	29.66 ± 4.08			73 (90.12)	67 (83.75)		
Unemployed/temporary workers	21 (13.04%)	28.48 ± 4.51			8 (9.88)	13 (16.25)		
Nature of work			− 1.357	0.177			1.352	0.245
Physical work	114 (70.81%)	29.22 ± 3.88			54 (66.67)	60 (75.00)		
Mental work	47 (29.19%)	30.19 ± 4.70			27 (33.33)	20 (25.00)		
Payment of medical expenses			0.515	0.607			0	0.993
Health insurance	159 (98.76%)	29.52 ± 4.14			1.352	0.245		
Self-pay	2 (1.24%)	28.00 ± 5.66			1.352	0.245		

Data were expressed as n (%), mean ± SD.

*T* t-tests, ***χ***^***2***^ Chi-square analysis, *F* ANOVA.

*P < 0.05, **P < 0.01.

### The association between psychological resilience, self-efficacy, social support, and PTSD

An analysis of the relationship between resilience and self-efficacy, social support, and PTSD (Table 2) showed that the high-resilience group had significantly higher self-efficacy and social support than the low-resilience group. PTSD in the high-resilience group was substantially lower. Bivariate analysis revealed that resilience was positively related to self-efficacy (*r* = 0.554, *P* < 0.01), social support and its dimensions (family support, friend support) (*r* = 0.432, 0.449, 0.343; *P* < 0.01), and PTSD and its dimensions (re-experience, avoidance/numbing, hyperarousal) (*r* =  − 0.476, − 0.274, − 0.241, − 0.420; *P* < 0.01).

**Table 2:** Statistical description of continuous variables and resilience correlation analysis.

Variables	Mean (SD)	High resilience	Low resilience	Resilience	
		Mean (SD)	Mean (SD)	r value	P value
Resilience	29.50 ± 4.158	32.90 ± 2.19	26.05 ± 2.50	1	0.009
General self-efficacy	29.677 ± 4.765	32.32 ± 3.58	27.20 ± 4.24	0.554	0.000**
Social support	60.118 ± 7.992	63.43 ± 7.23	53.70 ± 5.49	0.432	0.000**
Family support	20.012 ± 4.522	21.95 ± 3.92	17.39 ± 3.46	0.449	0.000**
Friends support	20.025 ± 4.189	21.22 ± 4.37	17.69 ± 3.11	0.343	0.000**
Other support	20.081 ± 4.155	20.25 ± 4.13	18.66 ± 3.58	− 0.003	0.973
Post-traumatic stress disorder	35.727 ± 8.309	29.84 ± 5.54	39.83 ± 7.84	− 0.476	0.000**
Re-experience	12.627 ± 4.337	10.68 ± 3.52	13.84 ± 3.79	− 0.274	0.000**
Avoidance/numbing	12.168 ± 3.308	11.11 ± 2.93	12.54 ± 3.24	− 0.241	0.000**
Hyperarousal	10.932 ± 4.697	8.14 ± 3.00	13.45 ± 4.46	− 0.420	0.000**

r value: Pearson or Spearman correlation coefficient.

*P < 0.05, **P < 0.01.

### Influencing factors

The regression model showed that resilience was significantly correlated with five different variables (Table 3) (Adjusted R2 = 0.514, *P* < 0.05). Monthly household income (β = 0.167, *P* <0.01), general self-efficacy (β = 0.397, *P* < 0.01), and social support (β = 0.225, *P* < 0.01) were positively associated with resilience, while PTSD (β =  − 0.284,* P* < 0.01) was negatively related to resilience. Logistic regression analysis with resilience as a categorical variable (Table 4) showed that general self-efficacy (OR 0.716, *P* < 0.01) and social support (OR 0.772, *P* < 0.01) were protective factors for psychological resilience. In contrast, PTSD (OR 1.278, *P* < 0.01) was a risk factor.

**Table 3:** Results of stepwise regression analysis.

	Unstandardized coefficients		Standardized coefficients	t	P	VIF	*R*^*2*^	Adjusted *R*^*2*^	*F*
	B	Standard error	Beta						
Constant	12.152	2.905	–	4.183	0.000**	–	0.530	0.514	*F* (5,155) = 34.898, *P* < 0.05*
Monthly household income	1.128	0.374	0.167	3.021	0.003**	1.011			
General self-efficacy	0.345	0.051	0.397	6.823	0.000**	1.116			
Social support	0.117	0.031	0.225	3.818	0.000**	1.145			
Posttraumatic stress disorder	− 0.142	0.029	− 0.284	− 4.831	0.000**	1.139			

Data are expressed as regression coefficients (β) ± standard error.

*P < 0.05, **P < 0.01.

**Table 4:** Univariate and multifactor analysis of factors influencing psychological resilience.

Variables	Univariate analysis		Multivariate Analysis	
	OR (95% CI)	P value	OR (95% CI)	P value
Education				
Primary school	4.600 (1.360–15.554)	0.014*	10.522 (0.378–294.797)	0.165
High or secondary school	2.747 (0.923–8.174)	0.069	4.539 (0.254–81.138)	0.304
College or university	Reference	–		
Monthly household income				
< 5000	2.925 (1.005–8.516)	0.049*	12.597 (0.984–161.193)	0.051
5,000–10,000	2.812 (1.280–6.179)	0.010*	2.866 (0.522–15.726)	0.225
> 10,000	Reference	–		
State of residence				
Living with others	2.853 (1.112–7.318)	0.029*	3.296 (0.381–28.502)	0.279
Living alone	Reference	–		
General self-efficacy	0.729 (0.658–0.809)	0.000**	0.716 (0.589–0.870)	0.001**
Posttraumatic stress disorder	1.354 (1.227–1.495)	0.000**	1.278 (1.077–1.515)	0.005**
Hyperarousal	1.479 (1.302–1.679)	0.000**	1.246 (0.968–1.605)	0.087
Social support	0.783 (0.725–0.845)	0.000**	0.772 (0.635–0.938)	0.009**
Family support	0.727 (0.653–0.808)	0.000**	0.883 (0.705–1.105)	0.276
Friends support	0.787 (0.717–0.865)	0.000**	0.977 (0.793–1.203)	0.826

OR odds ratio, CI confidence interval .

*P < 0.05, **P < 0.01.

**Figure 1:**
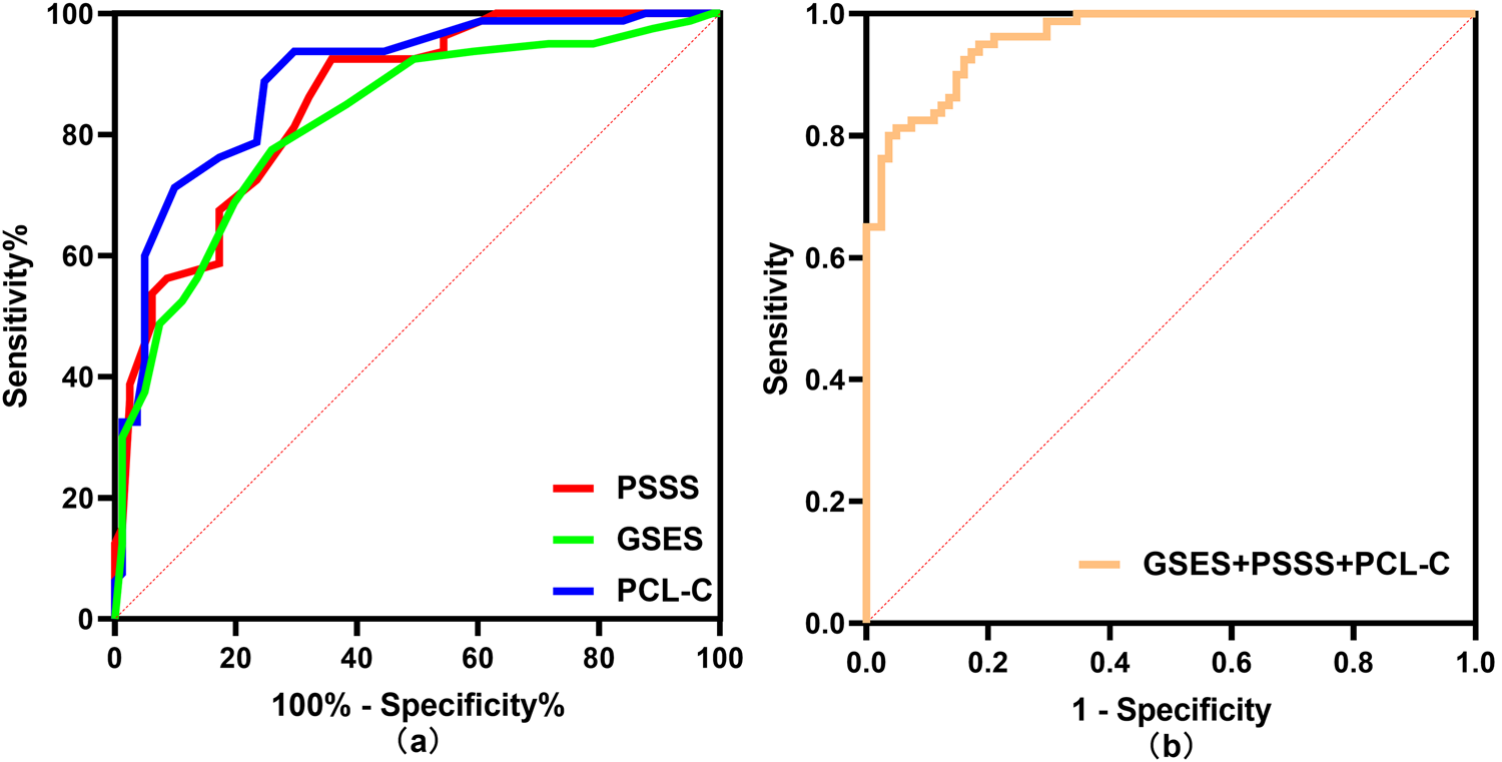
ROC curves of different variables to predict low resilience.

ROC analysis showed that the combination of the three variable scores had the most significant AUC of 0.961 (*P* < 0.01) (Fig. 1b), compared to GSES (AUC = 0.822), PCL-C (AUC = 0.889), and PSSS (AUC = 0.855) alone (Fig. 1a), with some predictive values for high and low levels of resilience (all *P* < 0.01). The optimal cut-off values, sensitivity, and specificity of the three variables are shown in Table 5.

**Table 5:** Optimal cut-off value, sensitivity, and specificity of GSES, PSSS, and PCL-C scores for predicting low psychological resilience.

Variables	AUC	95% CI	P-value	Yoden index	Optimal cut-off value	Sensitivity (%)	Specificity (%)
General self-efficacy (GSES)	0.822	0.758–0.887	0.001**	0.516	30	77.50	74.07
Social support (PSSS)	0.855	0.799–0.912	0.001**	0.567	60	92.50	64.20
PTSD check list(PCL-C)	0.889	0.838–0.940	0.001**	0.641	31	93.75	70.37
GSES + PSSS + PCL-C	0.961	0.937–0.985	0.001**	0.765	-	95.00	81.48

*P < 0.05, **P < 0.01.

*OR *odds ratio, *CI *confidence interval.

*P < 0.05, **P < 0.01.

## Discussion

### Current status of resilience in young and middle-aged patients with first myocardial infarction

This study discovered that the resilience level in young and middle-aged patients with their first AMI was 29.50 ± 4.158, with the scores of high and low resilience groups being 32.90 ± 2.19 and 26.05 ± 2.50, respectively. Self-efficacy, social support, and PTSD were significantly better in the high-resilience group than in the low-resilience group. The first symptom of AMI is generally severe chest pain, with rapid onset and progression, and the mental stimulation and psychological stress generated during the process of being rushed to the emergency room are enormous. Psychological resilience and its protective factors can assist individuals in coping with stress, improving their mental health, and maintaining patients’ physical and psychological health^29^. Patients with low resilience should be given more social attention because patients’ psychological adjustment disorders, such as self-perceived burden, reduced self-care ability, and a lack of social roles can lead to patients’ inability to cope with the disease. These changes weaken their active healthcare-seeking behaviors, which is detrimental to their recovery^30^. According to this study, monthly household income, self-efficacy, and social support all positively influence resilience, whereas PTSD was negatively associated with resilience. These factors displayed 51.4% of the variance in resiliency among first-time AMI patients (Supplementary Information).

### Post-traumatic stress disorder and resilience

It has been reported that positive PTSD adversely affects cardiac events and quality of life^31,32^. Individuals with high psychological resilience have a low prevalence of PTSD^33^, and PTSD is negatively associated with resilience in a survey of burn patients^34^. This study also revealed a significant negative association with lower resilience scores in participants with positive PTSD symptoms (β =  − 0.284, *P* < 0.01) and a 1.143-fold increase in the probability of PTSD in low resilience participants than high resilience participants (*OR* 1.278, *P* < 0.01). Therefore, a high level of psychological resilience improves patients’ adaptability to adversity and promotes disease regression.

Cardiovascular diseases such as acute myocardial infarction are considered “exclusive diseases” of the elderly. Therefore, accepting the diagnosis of a sudden and severe disease, such as acute myocardial infarction, is challenging for young patients psychologically, resulting in pessimistic behavior about the prognosis and increasing their stress response. Patients may develop PTSD due to the severe pain and psychological stress caused by a sudden AMI. A review showed a higher incidence of PTSD in younger acute coronary syndrome (ACS) patients^35^. This study found that the optimal cut-off value for predicting low resilience was 31, less than the positive cut-off value of 38, currently recommended for use in the literature^36^. This finding could be related to the type of disease and sample population in this study. This study highlighted the importance of nurses identifying high-risk PTSD patients as early as possible and implementing effective coping strategies for PTSD. In addition, nurses must pay special attention to the families of patients with low education levels, self-pay medical expenses, and low self-care levels and assist them in using appropriate coping strategies and social support systems to better avoid traumatic and stressful events^37^.

### Self-efficacy and resilience

This study found self-efficacy to be significantly and positively related to resilience (β = 0.397, *P* < 0.01), consistent with other diseased populations, such as stroke, breast cancer, and burn injury patients^38–40^. Self-efficacy can improve the quality of life for AMI patients by boosting their self-confidence and positive attitude about dealing with the disease^41^. This study validated self-efficacy’s protective effect on resilience (*OR* 0.716, *P* < 0.01). An analysis of self-efficacy in elderly AMI patients showed that the mean self-efficacy score after PCI was 21.56 ± 9.66^12^. In contrast, the mean self-efficacy score of young and middle-aged AMI patients after emergency PCI in this study was 29.677 ± 4.765, higher than in previous studies. This difference is due to the loss of the labor force, fewer social roles, reduced confidence in treatment (due to underlying diseases), and a lower sense of self-worth in the elderly. Middle-aged and young people, on the other hand, are at a critical juncture in their careers, have higher expectations for disease recovery, and are willing to fully cooperate with treatment, influencing their feelings positively.

In addition, this study established ROC prediction thresholds for variables associated with low resilience. Therefore, a GSES score < 30 predicted low resilience with a sensitivity and specificity of 77.50% and 74.07%, respectively, indicating the need to prevent low resilience by enhancing self-efficacy. Consequently, in clinical nursing, nurses should focus on cultivating and improving patients’ self-efficacy with myocardial infarction to encourage patients to maintain a positive psychological state during disease treatment, thereby increasing their psychological resilience.

### Social support and resilience

Younger AMI patients with lower levels of social support have worse mental health, quality of life, and depressive symptoms^42^. However, no research has explored the relationship between social support and resilience in young and middle-aged AMI survivors. Some studies in other populations have discovered a link between social support and resilience^38,43^. Young and middle-aged patients are generally unable to fulfill their established family and social roles following the illness and are concerned about the disease’s impact on their prognosis. After their first AMI, young and middle-aged patients showed a moderately positive relationship between resilience and social support (*β* = 0.225, *P* < 0.01), implying that positive social support can reduce negative stress-related emotions by encouraging the expression of their emotions, improving their ability to cope with the disease^44^, helping them overcome their fear about their condition, and boost their resilience^45^. Consequently, nurses should prioritize patients with low social support in their work, assist patients in changing their negative perceptions, actively seek outside consent, and encourage family members to provide more care and encouragement to patients.

### Monthly household income and resilience

Furthermore, this study showed that high resilience was predicted by higher monthly household income (> 10,000 RMB), contradicting previous research. However, no statistically significant difference has been observed between economic status and resilience in patients with oral and breast cancer^46,47^. This study hypothesized that a higher monthly household income might indicate a greater ability and capital to deal with difficulties and adversity as an important safeguard against misfortune. Low-income patients, on the other hand, bear a heavier household burden, resulting in lower resilience.

## Limitations

In this study, there were three limitations. First, an analysis was conducted on the relationship between self-efficacy, social support, PTSD, and resilience only; other potential factors that might influence resilience need further research. Second, determining the causal relationship between resilience and its influencing factors is challenging to determine using this single-center cross-sectional study; other studies should validate the conclusions of this study. Third, although the sample size met statistical needs and presented significant results, its small size may have affected the generalisability of the results. Future studies should consider larger sample sizes to strengthen the reliability of the findings.

## Conclusion

Among young and middle-aged AMI patients undergoing emergency PCI, PTSD was the strongest predictor and risk factor of psychological resilience, followed by social support. Improving resilience is essential for improving individuals’ positive coping with illness after AMI. This study contributes to the development of resilience-building interventions.

### Supplementary Information

Supplementary Information.

## Data Availability

Data will be shared on request. If someone wants to request the data from this study, they can contact Jinju Wang.

## Abbreviations

AMI: Acute myocardial infarction
PCI: Percutaneous coronary intervention
PTSD: Post-traumatic stress disorder
ROC: Receiver operating curve
CCU: Coronary care unit
CD-RISC-10: Connor–Davidson resilience scale 10
GSES: General self‐efficacy scale
PSSS: Perceived social support scale
PCL-C: The PTSD check list-civilian version
