# Supplementary material for: Resilience and its influencing factors after emergency percutaneous coronary intervention in young and middle-aged patients with first acute myocardial infarction
Source: Sci Rep. 2024 Apr 25;14:9507. doi: 10.1038/s41598-024-59885-9 (PMC11045793; doi:10.1038/s41598-024-59885-9)
Supplement: Supplementary file 1 — Supplementary Information. [file 41598_2024_59885_MOESM1_ESM.pdf]

Resilience and Its Influencing Factors After Emergency Percutaneous Coronary Intervention in Young and Middle-Aged Patients with First Acute Myocardial Infarction

Author Information：

Jinju Wang※ 997713144@qq.com

Yafeng Wu※ 479013690@qq.com

Juanjuan Zhou※ 261347671@qq.com

Shaoman Li 1521593769@qq.com

Liping She sheliping80@163.com

Department of Cardiovascular, Nanjing Hospital, Nanjing Medical University (Nanjing First Hospital) Nanjing 210000, China

General Information Survey Questionnaire

1. Name and Hospital ID?

    ____

2. Age?

    <45   45-60

3. Gender?

    Male   Female

4. Do you have a spouse?

    Yes   No

5. Employment status

    Employed   Unemployed   Retired

6. What is the nature of your work (including before retirement)?

    Physical   Mental

7. Are you covered by medical insurance?

    Yes   No

8. What is your educational level?

    Elementary school or below

    Junior high school/Technical secondary school or high school

    College or above

9. What is your total monthly income?

    Below 5000   5000-10000   Above 10000

10. Do you live alone?

    Yes   No (living with family or friends)

11. Your current place of residence?

    City   County town   Rural area

CD-RISC-10 Scale

For each of the following statements, please indicate how much you agree or disagree with it, using the following scale:

1. I am able to adapt to change.

0 (Very non-conforming)

1 (Non-conforming)

2 (Neutral)

3 (Conforming)

4 (Very conforming)

2. I can deal with whatever comes my way.

0 (Very non-conforming)

1 (Non-conforming)

2 (Neutral)

3 (Conforming)

4 (Very conforming)

3. I try to see the humorous side of things when I am faced with problems.

0 (Very non-conforming)

1 (Non-conforming)

2 (Neutral)

3 (Conforming)

4 (Very conforming)

4. Having to cope with stress makes me stronger.

0 (Very non-conforming)

1 (Non-conforming)

2 (Neutral)

3 (Conforming)

4 (Very conforming)

5. I tend to bounce back after illness, injury, or other hardships.

0 (Very non-conforming)

1 (Non-conforming)

2 (Neutral)

3 (Conforming)

4 (Very conforming)

6. I believe I can achieve my goals, even if there are obstacles.

0 (Very non-conforming)

1 (Non-conforming)

2 (Neutral)

3 (Conforming)

4 (Very conforming)

7. Under pressure, I stay focused and think clearly.

0 (Very non-conforming)

1 (Non-conforming)

2 (Neutral)

3 (Conforming)

4 (Very conforming)

8. I am not easily discouraged by failure.

0 (Very non-conforming)

1 (Non-conforming)

2 (Neutral)

3 (Conforming)

4 (Very conforming)

9. I think of myself as a strong person when dealing with life's challenges and difficulties.

0 (Very non-conforming)

1 (Non-conforming)

2 (Neutral)

3 (Conforming)

4 (Very conforming)

10. I am able to handle unpleasant or painful feelings like sadness, fear, and anger.

0 (Very non-conforming)

1 (Non-conforming)

2 (Neutral)

3 (Conforming)

4 (Very conforming)

General Self-Efficacy Scale (GSES)

1. I can always manage to solve difficult problems if I try hard enough.

1 (Completely incorrect)

2 (Mostly incorrect)

3 (Mostly correct)

4 (Completely correct)

2. If someone opposes me, I can find the means and ways to get what I want.

1 (Completely incorrect)

2 (Mostly incorrect)

3 (Mostly correct)

4 (Completely correct)

3. It is easy for me to stick to my aims and accomplish my goals.

1 (Completely incorrect)

2 (Mostly incorrect)

3 (Mostly correct)

4 (Completely correct)

4. I am confident that I could deal efficiently with unexpected events.

1 (Completely incorrect)

2 (Mostly incorrect)

3 (Mostly correct)

4 (Completely correct)

5. Thanks to my resourcefulness, I know how to handle unforeseen situations.

1 (Completely incorrect)

2 (Mostly incorrect)

3 (Mostly correct)

4 (Completely correct)

6. I can solve most problems if I invest the necessary effort.

1 (Completely incorrect)

2 (Mostly incorrect)

3 (Mostly correct)

4 (Completely correct)

7. I can remain calm when facing difficulties because I can rely on my coping abilities.

1 (Completely incorrect)

2 (Mostly incorrect)

3 (Mostly correct)

4 (Completely correct)

8. When I am confronted with a problem, I can usually find several solutions.

1 (Completely incorrect)

2 (Mostly incorrect)

3 (Mostly correct)

4 (Completely correct)

9. If I am in trouble, I can usually think of a solution.

1 (Completely incorrect)

2 (Mostly incorrect)

3 (Mostly correct)

4 (Completely correct)

10. I can usually handle whatever comes my way.

1 (Completely incorrect)

2 (Mostly incorrect)

3 (Mostly correct)

4 (Completely correct)

Participants rate each item based on their agreement with the statement, with higher scores indicating greater self-efficacy.

Perceived Social Support Scale (PSSS)

1. There is a special person who is around when I am in need.

1 (Strongly Disagree)

2 (Disagree)

3 (Mildly Disagree)

4 (Neutral)

5 (Mildly Agree)

6 (Agree)

7 (Strongly Agree)

2. There is a special person with whom I can share my joys and sorrows.

1 (Strongly Disagree)

2 (Disagree)

3 (Mildly Disagree)

4 (Neutral)

5 (Mildly Agree)

6 (Agree)

7 (Strongly Agree)

3. My family really tries to help me.

1 (Strongly Disagree)

2 (Disagree)

3 (Mildly Disagree)

4 (Neutral)

5 (Mildly Agree)

6 (Agree)

7 (Strongly Agree)

4. I get the emotional help and support I need from my family.

1 (Strongly Disagree)

2 (Disagree)

3 (Mildly Disagree)

4 (Neutral)

5 (Mildly Agree)

6 (Agree)

7 (Strongly Agree)

5. I have a special person who is a real source of comfort to me.

1 (Strongly Disagree)

2 (Disagree)

3 (Mildly Disagree)

4 (Neutral)

5 (Mildly Agree)

6 (Agree)

7 (Strongly Agree)

6. My friends really try to help me.

1 (Strongly Disagree)

2 (Disagree)

3 (Mildly Disagree)

4 (Neutral)

5 (Mildly Agree)

6 (Agree)

7 (Strongly Agree)

7. I can count on my friends when things go wrong.

1 (Strongly Disagree)

2 (Disagree)

3 (Mildly Disagree)

4 (Neutral)

5 (Mildly Agree)

6 (Agree)

7 (Strongly Agree)

8. I can talk about my problems with my family.

1 (Strongly Disagree)

2 (Disagree)

3 (Mildly Disagree)

4 (Neutral)

5 (Mildly Agree)

6 (Agree)

7 (Strongly Agree)

9. I have friends with whom I can share my joys and sorrows.

1 (Strongly Disagree)

2 (Disagree)

3 (Mildly Disagree)

4 (Neutral)

5 (Mildly Agree)

6 (Agree)

7 (Strongly Agree)

10. There is a special person in my life who cares about my feelings.

1 (Strongly Disagree)

2 (Disagree)

3 (Mildly Disagree)

4 (Neutral)

5 (Mildly Agree)

6 (Agree)

7 (Strongly Agree)

11. My family is willing to help me make decisions.

1 (Strongly Disagree)

2 (Disagree)

3 (Mildly Disagree)

4 (Neutral)

5 (Mildly Agree)

6 (Agree)

7 (Strongly Agree)

12. I can talk about my problems with my friends.

1 (Strongly Disagree)

2 (Disagree)

3 (Mildly Disagree)

4 (Neutral)

5 (Mildly Agree)

6 (Agree)

7 (Strongly Agree)

Participants rate each item based on their agreement with the statement, with higher scores indicating greater perceived social support.

PTSD Checklist-Civilian Version (PCL-C)

1. Repeated, disturbing memories, thoughts, or images of a stressful experience from the past?

1 (Did not occur)

2 (A little bit)

3 (Moderately)

4 (Quite a bit)

5 (Extremely severe)

2. Repeated, disturbing dreams of a stressful experience from the past?

1 (Did not occur)

2 (A little bit)

3 (Moderately)

4 (Quite a bit)

5 (Extremely severe)

3. Suddenly acting or feeling as if a stressful experience were happening again (as if you were reliving it)?

1 (Did not occur)

2 (A little bit)

3 (Moderately)

4 (Quite a bit)

5 (Extremely severe)

4. Feeling very upset when something reminded you of a stressful experience from the past?

1 (Did not occur)

2 (A little bit)

3 (Moderately)

4 (Quite a bit)

5 (Extremely severe)

5. Having physical reactions (e.g., heart pounding, trouble breathing, sweating) when something reminded you of a stressful experience from the past?

1 (Did not occur)

2 (A little bit)

3 (Moderately)

4 (Quite a bit)

5 (Extremely severe)

6. Avoiding thinking about or talking about a stressful experience from the past or avoiding having feelings related to it?

1 (Did not occur)

2 (A little bit)

3 (Moderately)

4 (Quite a bit)

5 (Extremely severe)

7. Avoiding activities or situations because they remind you of a stressful experience from the past?

1 (Did not occur)

2 (A little bit)

3 (Moderately)

4 (Quite a bit)

5 (Extremely severe)

8. Trouble remembering important parts of a stressful experience from the past?

1 (Did not occur)

2 (A little bit)

3 (Moderately)

4 (Quite a bit)

5 (Extremely severe)

9. Loss of interest in activities that you used to enjoy?

1 (Did not occur)

2 (A little bit)

3 (Moderately)

4 (Quite a bit)

5 (Extremely severe)

10. Feeling distant or cut off from other people?

1 (Did not occur)

2 (A little bit)

3 (Moderately)

4 (Quite a bit)

5 (Extremely severe)

11. Feeling emotionally numb or being unable to have loving feelings for those close to you?

1 (Did not occur)

2 (A little bit)

3 (Moderately)

4 (Quite a bit)

5 (Extremely severe)

12. Feeling as if your future will somehow be cut short?

1 (Did not occur)

2 (A little bit)

3 (Moderately)

4 (Quite a bit)

5 (Extremely severe)

13. Trouble falling or staying asleep?

1 (Did not occur)

2 (A little bit)

3 (Moderately)

4 (Quite a bit)

5 (Extremely severe)

14. Feeling irritable or having angry outbursts?

1 (Did not occur)

2 (A little bit)

3 (Moderately)

4 (Quite a bit)

5 (Extremely severe)

15. Having difficulty concentrating?

1 (Did not occur)

2 (A little bit)

3 (Moderately)

4 (Quite a bit)

5 (Extremely severe)

16. Being "super alert" or watchful or on guard?

1 (Did not occur)

2 (A little bit)

3 (Moderately)

4 (Quite a bit)

5 (Extremely severe)

17. Feeling jumpy or easily startled?

1 (Did not occur)

2 (A little bit)

3 (Moderately)

4 (Quite a bit)

5 (Extremely severe)

Participants rate each item based on how much they have been bothered by that symptom in the past month, with higher scores indicating greater severity of PTSD symptoms.
